# A telehealth program for CPAP adherence reduces labor and yields similar adherence and efficacy when compared to standard of care

**DOI:** 10.1007/s11325-015-1298-4

**Published:** 2016-01-11

**Authors:** Dominic Munafo, William Hevener, Maureen Crocker, Leslee Willes, Sarah Sridasome, Ma’an Muhsin

**Affiliations:** Sleep Data, 4420 Hotel Ct #240, San Diego, CA 92108 USA; ResMed Science Center, ResMed Corp, 9001 Spectrum Center Blvd., San Diego, CA USA; Willes Consulting Group, Inc., 1327 Walnutview Drive, Encinitas, San Diego, CA 92024 USA

**Keywords:** Obstructive sleep apnea, Telehealth, Continuous positive airway pressure, Adherence, Compliance coaching, Labor

## Abstract

**Purpose:**

This study evaluated the effectiveness and coaching labor requirements of a web-based automated telehealth (TH) messaging program compared with standard of care (SOC) in newly diagnosed patients with obstructive sleep apnea (OSA).

**Methods:**

In this non-blinded, multicenter, prospective study, all patients were started on continuous positive airway pressure (CPAP) with heated humidification and a wireless modem. They all received standardized CPAP education and setup. Patients in the TH group (*n* = 58) received an automated series of text messages and/or e-mails that were triggered by preset conditions. Patients in the SOC group (*n* = 64) received scheduled calls on days 1, 7, 14, and 30. Additional contacts were allowed for patients in both groups as deemed clinically necessary. Coaching labor was calculated by totaling the number and type of patient contacts and assigning historical time values.

**Results:**

One hundred twenty-two patients were included in the final analysis. There were no statistically significant differences between the TH and SOC groups for Medicare adherence (83 vs. 73 %), daily CPAP usage (5.1 ± 1.9 h vs. 4.7 ± 2.1 h), CPAP efficacy (mean residual apnea-hypopnea index (3.0 ± 4.1/h vs. 2.8 ± 3.8/h), or change in Epworth Sleepiness Scale score (−5.8 ± 5.5 vs. –5.1 ± 5.9), although all trends favored the TH group. There was, however, a significant reduction in the number of minutes coaching required per patient in the TH vs. SOC group (23.9 ± 26 vs. 58.3 ± 25, 59 % reduction; *p* < 0.0001).

**Conclusions:**

Use of a web-based telehealth program for CPAP adherence coaching significantly reduced the coaching labor requirement compared with SOC, while maintaining similar adherence and effectiveness.

## Introduction

Obstructive sleep apnea (OSA) is the most common form of sleep-disordered breathing (SDB) and is typified by repetitive complete or partial collapse of the upper airway during sleep. The prevalence of OSA has been increasing steadily, in part due to the rise in obesity, with recent estimates suggesting that it affects ≥13 % of men and 6 % of women [1, 2]. This represents a considerable growth rate of 14–55 % over the past two decades [2]. OSA has been associated with numerous co-morbidities including hypertension, congestive heart failure, stroke, diabetes mellitus, and an increased risk of motor vehicle accidents [3–10]. OSA places a substantial financial burden on the healthcare system, with the cost of untreated OSA estimated to be US$67–165 billion [11].

First-line therapy for OSA is continuous positive airway pressure (CPAP), which improves symptoms including excessive daytime sleepiness, fatigue, memory impairment, and depressed mood [12, 13]. Long-term adherence with CPAP therapy has also been shown to improve blood pressure and cardiovascular morbidity and mortality [14]. Conversely, OSA symptoms return when CPAP therapy is discontinued [15]. Unfortunately, CPAP therapy can take some time to get used to, and long-term adherence with therapy can be challenging. Historically, CPAP adherence rates have been ≈50 % and when applying Medicare’s definition of adherence (≥4 h CPAP usage each night for 70 % of nights during a 30 consecutive-day period anytime during the first 90 days of initial usage), the rate has been reported to be as low as 46 % [14, 16, 17]. However, more recent reports have documented adherence rates of 60–85 % when more intensive follow-up and educational protocols are used [18].

Lack of adherence with long-term regimens is an ongoing challenge for both patients and healthcare providers. It has been estimated that non-adherence results in annual costs of $290 billion in the USA alone [19]. World Health Organization (WHO) data indicate that patients with chronic diseases only adhere to their doctor’s long-term recommended therapy 50 % of the time [19]. Such data led to the conclusion that “interventions aimed at improving adherence would provide a significant positive return on investment through primary prevention (of risk factors) and secondary prevention of adverse health outcomes” [19].

Telehealth (TH) has been defined as the delivery of health-related services and information via telecommunications technologies. The development and increasing availability of TH technology offers the opportunity to provide quality care while effectively managing costs. Chronic diseases require long-term commitment of resources for a successful therapy program and, as such, are an excellent area to apply TH technology.

U-Sleep (ResMed Corp) is a TH program that uses a multi-media approach to contact patients about their CPAP use. When usage falls, e-mail and text messages are sent to encourage patients to use CPAP more regularly. Electronic messages are also sent to the patient’s clinician, with triggered alerts to further assist in CPAP management. Clinicians also remotely monitor their patients’ progress using U-Sleep technology via metrics including daily usage, apnea-hypopnea index (AHI) values, and leak. There are currently no published clinical research studies on use of U-Sleep technology as a tool to facilitate management of OSA patients.

This study compared the U-Sleep program with a standard-of-care (SOC) program in patients with OSA receiving CPAP therapy for the first time. The primary objective was to compare adherence rates and healthcare professional (HCP) time (resource use) in the two groups.

## Methods

This randomized, prospective, non-blinded study was conducted by Sleep Data Holdings, LLC, a Joint Commission on Accreditation of Healthcare Organizations-accredited CPAP durable medical equipment provider in Southern California, USA. The study protocol, informed consent form, and Health Insurance Portability and Accountability Act (HIPAA) form were approved by the Chesapeake Institutional Review Board. Informed consent was obtained from all individual participants included in the study.

### Subjects

Patients were recruited and evaluated at two Sleep Data locations (San Diego County) in March–August 2013. Participants were consecutive patients referred to receive CPAP for OSA. Inclusion criteria were age 18–80 years, CPAP-naïve, confirmed OSA (AHI 5–70/h) diagnosis based on polysomnography (PSG) or home sleep test. In addition, patients were required to have access to and be able to utilize, communication technology (text messaging, e-mail). Exclusion criteria were prominent central apnea (>20 %), claustrophobia, current use of mandibular repositioning device, or other OSA therapy. A simple randomization scheme was used to allocate patients to CPAP treatment plus SOC or TH.

### CPAP therapy

At baseline, all patients had a 1-h education session with a respiratory therapist (RRT) about OSA and its consequences, proper use and maintenance of the CPAP device and mask, and therapy expectations. All patients were provided with a fixed or auto CPAP device, heated humidifier, modem, and mask interface (S9 Elite, S9 AutoSet, H5i heated humidifier; ResMed Corp.). Patients saw an RRT at all clinic visits; telephone follow-up was performed by registered PSG technicians (RPSGT).

### Adherence monitoring

**Fig. 1:**
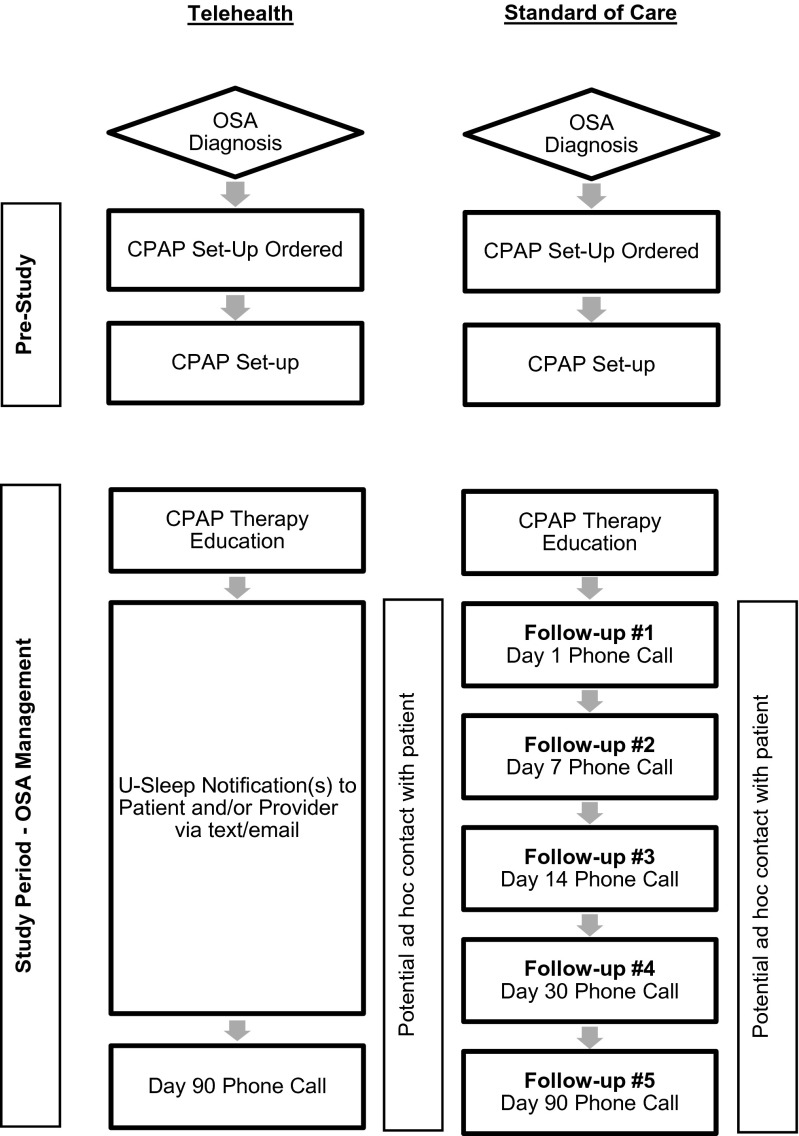
Study design. *CPAP* continuous positive airway pressure, *OSA* obstructive sleep apnea

Patients randomized to SOC were dispensed a CPAP device on Day 0, then contacted via phone on Days 1, 7, 14, 30, and 90 (Fig. 1). CPAP usage and efficacy data were tracked via the wireless modem attached to the CPAP machine. Modem data were accessed via ResMed’s EasyCare Online (ECO) platform. Sleep Data SOC procedures include frequent phone calls and return clinic visits as necessary.

Patients in the TH group were dispensed a CPAP device on Day 0, along with a pamphlet about U-Sleep, which was used to monitor adherence. U-Sleep is a secure, HIPAA-compliant, web-based application that is designed to receive CPAP device data and message patients and providers via text and/or e-mail based on a customizable set of rules. At the time of set up, patients were encouraged to log-in to the U-Sleep website from home so that they could follow their therapy. Sleep Data study staff were trained to set up and use the software, which was provided to patients at no charge. Initial patient contacts were triggered by ≥1 of five intervention points based on metrics (AHI, leak, therapy hours) (Table 1). After initial contact, subsequent contacts were in response to an automated message or based on clinical judgment. All TH patients received a final phone call on day 90. All patients were contacted at day 90 and asked to rate how well the follow-up program had met their expectations (on a scale from 1 to 5).

**Table 1:** U-Sleep notification triggers

Trigger	To whom	Method
No CPAP data for 2 consecutive days	Patient and provider	E-mail and/or text
CPAP usage <4 h for 3 consecutive nights	Patient and provider	E-mail and/or text
Median mask leak >24 L/min for 2 consecutive days	Provider	E-mail
AHI >15/h for 5 consecutive days	Provider	E-mail
CPAP usage met Medicare criteria for adherence	Patient and provider	E-mail and/or text

In both groups, all phone contacts were performed by an RPSGT from Sleep Data using a standardized script. Additional telephone contacts or clinic visits were scheduled as deemed clinically necessary by the RPSGT for evaluation of issues such as high mask leak, appropriate pressure settings, AHI elevation, and daily usage. Tasks undertaken at clinic visits included mask refit, patient re-education and training with an RRT, provision of a copy of CPAP therapy data downloads, and machine performance evaluations.

### Resource use

The total number of contacts was recorded. Contacts were categorized as contact attempt (e.g., staff left a phone message with patient to follow up; 4 min), phone contact (RPSGT communication with patient; 9 min), and clinic visits (e.g., mask refitting and education performed by an RRT; 30 min). Times assigned to each contact were determined based on a systematic review of Sleep Data’s clinical experience.

### Study endpoints

The primary endpoints were CPAP adherence (use for ≥4 h/night on 70 % of nights during a 30 consecutive-day period anytime during the first 90 days of initial usage) and resource use. The adherence rate was the percentage of patients who achieved Medicare adherence. Whether patient adherence with CPAP in the TH group was superior to the published average of 50 % [13] was also determined.

### Sample size

Equivalence in CPAP adherence was defined as a <20 % difference in rates between the SOC and TH groups. Assuming an expected adherence rate of 70 % (based on historical Sleep Data rates), with a power of ≥80 % (one-tailed alpha 0.05) to detect a ≥20 % difference, the enrolment target was 69 patients/group [20]. For comparing adherence in the TH group to the global average, a sample size of 37 patients/group would have ≥80 % power (one-tailed alpha 0.05) to detect a difference of ≥20 %, assuming an expected adherence rate for the TH group of 70 % and a published average of 50 %.

### Statistical analysis

Primary endpoint analyses were generated for the intention-to-treat (ITT) and completed cases (CC) populations. The ITT population included all randomized patients except two who withdrew consent. Patients with no compliance data, and one patient who never enrolled in the U-Sleep program, were considered non-adherent to CPAP; for those lost-to-follow-up, adherence results for the last available assessment were used. The CC population included patients who completed the study according to the protocol. Additional analyses were conducted in the CC population without imputation for missing values.

Equivalence in CPAP adherence between the two treatment groups was tested using a two-sided 95 % confidence interval (CI) for the difference in proportions. The null hypothesis is rejected and equivalence established if the upper limit of the CI is ≤20 %. The superiority hypothesis (TH adherence vs. published average adherence) was tested using a two-sided exact binomial test of the TH adherence proportion compared to typical published average adherence; a two-sided 95 % CI was calculated for the TH adherence proportion.

Baseline characteristics and 90-day evaluations were compared between treatment groups using Student’s *t* test for continuous variables and chi-square or Fisher’s exact tests for categorical variables, as appropriate. Wilcoxon rank sum tests were employed when a non-parametric test of continuous variables was warranted. *P* values of <0.05 were considered statistically significant. Analyses were generated using SAS software, version 9.2 or greater.

## Results

**Fig. 2:**
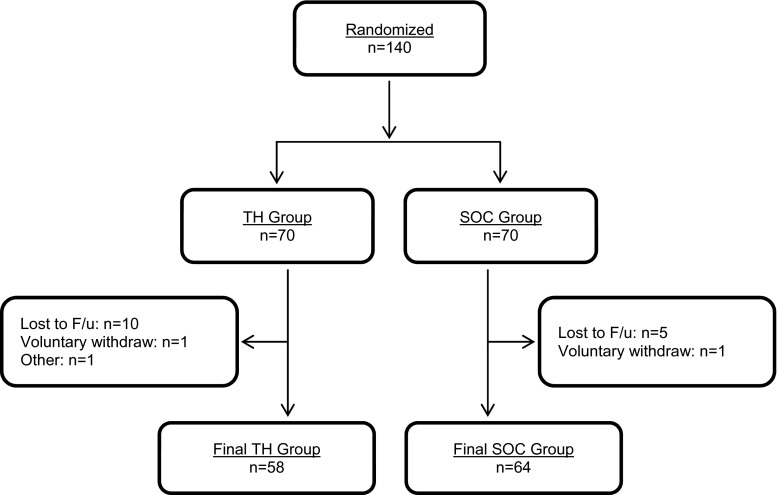
Patient flow through the study. *TH* telehealth, *SOC* standard of care

All approached patients consented to participate (*n* = 140); 18 patients did not complete the 90-day study (16 lost to follow-up, 2 withdrew consent) (Fig. 2). The ITT and CC populations comprised 138 and 122 patients, respectively. Baseline characteristics were similar in the TH and SOC groups (Table 2). CPAP therapy had similar effectiveness in both treatment groups, based on residual AHI and Epworth Sleepiness scale score.

**Table 2:** Baseline patient characteristics

Characteristic	TH group (*N* = 69)	SOC group (*N* = 69)	*p* value
Age, years (mean±SD^a^)	52.3 ± 10.6	50.0 ± 11.7	0.23
Male sex, *n* (%)	50/69 (72.5)	45/69 (65.2)	0.36
Body mass index, kg/m^2^ (mean ± SD)	33.5 ± 8.2	32.9 ± 7.1	0.63
Ethnicity, *n* (%)	(68)	(61)	
Caucasian	49/68 (72.1)	50/61 (82.0)	0.34
Hispanic	11/68 (16.2)	4/61 (6.6)	
African-American	3/68 (4.4)	2/61 (3.3)	
Asian	5/68 (7.4)	4/61 (6.6)	
Other	0 (0)	1/61 (1.6)	
ESS^b^ score	(*n* = 66)	(*n* = 68)	
Mean ± SD	10.9 ± 4.7	10.2 ± 5.7	0.42
Median	11.0	10.0	
AHI^c^/h	(*n* = 69)	(*n* = 69)	
Mean ± SD	33.4 ± 24.5	27.4 ± 18.0	0.23
Median	27.2	23.2	
PAP device, *n* (%)			
S9 Elite	59/69 (85.5)	58/69 (84.1)	0.81
AutoSet	10/69 (14.5)	11/69 (15.9)	
Mask type, *n* (%)			
FFM^d^	20/68 (29.4)	20/69 (29.0)	0.90
Nasal mask	39/68 (57.4)	38/69 (55.1)	
Nasal pillow	9/68 (13.2)	11/69 (15.9)	
Device therapy setting, cmH_2_O	(*n* = 58)	(*n* = 58)	
Mean ± SD (*n*)	10.6 ± 2.5	10.5 ± 2.0	0.81
Median	10.0	10.0	

^a^Standard deviation

^b^Epworth Sleepiness Scale

^c^Apnea-hypopnea index

^d^Full face mask

### Primary endpoint (ITT)

Medicare adherence rates were 69.6 % in the TH group and 68.1 % in the SOC group (*p* = 0.85). The upper 95 % confidence limit of the difference between groups was 14.0 %, meeting predefined equivalence criteria. The Medicare adherence rate in the TH group was 69.6 % (95 % CI 57.3–80.1 %) compared with the published average of 50 % (*p* = 0.002).

### Primary endpoint (CC)

Adherence rates were excellent and similar in both groups (*p* = 0.22) (Table 3). The Medicare adherence rate in the TH group was 82.8 % (95 % CI 70.6–91.4 %), significantly higher than the published average of 50 % (*p* < 0.0001). The Medicare adherence rate in the SOC group was 73.4 %.

**Table 3:** Adherence results in the completed cases population

	TH	SOC	*p* value
Medicare adherence rates	*N* = 58	*N* = 64	
Patients achieving adherence in first 30 days, *n* (%)	36 (62.1)	34 (53.1)	0.40
Patients achieving adherence within 31–60 days, *n* (%)	10 (17.2)	8 (12.5)	
Patients achieving adherence within 61–90 days, *n* (%)	2 (3.5)	5 (7.8)	
Patients not achieving adherence during the study, *n* (%)	10 (17.2)	17 (26.6)	
Total patients achieving adherence at any time, *n* (%)	48 (82.8)	47 (73.4)	0.22
Average time to achieve adherence, days	(*n* = 48)	(*n* = 47)	
Mean±SD^a^ (range)	30.0 ± 12.2 (21–73)	34.0 ± 18.3 (21–83)	0.21
Usage	*N* = 58	*N* = 64	
Daily usage, h			
Mean ± SD (range)	5.1 ± 1.9 (0.4–9.1)	4.7 ± 2.1 (0.1–9.0)	0.24
Median	5.5	4.9	
Days CPAP used for >4 h, % patients			
Mean ± SD (range)	70.2 ± 26.7 (3.3–100)	63.3 ± 28.5 (0–98.9)	0.17
Median	77.8	71.1	

^a^Standard deviation

**Fig. 3:**
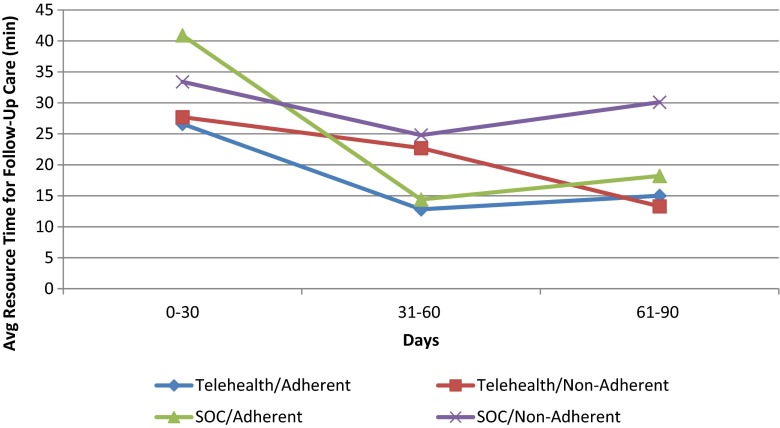
Average time spent on follow-up care by time from initiation of CPAP

The upper 95 % confidence limit for the difference in Medicare adherence rates was 5.2 %, again meeting equivalence criteria. The majority of patients in both groups achieved Medicare adherence within the first 30 days of CPAP initiation, and time to reach target adherence was similar in the two groups (Table 3 and Fig. 3). Mean CPAP usage and the percentage of days that patients used CPAP for ≥4 h were slightly, but not significantly, higher in the TH versus SOC group.

Multiple logistic regression analysis evaluating the effect of baseline characteristics on adherence rates showed that age and AHI at baseline were significantly associated with adherence. Adherent patients were significantly older than non-adherent patients and had a higher baseline AHI (Table 4).

**Table 4:** Characteristics of adherent and non-adherent patients

	Adherent	Non-adherent	*p* value^a^
Age, years—mean ± SD^b^ (range) [*n*]			
TH	52.2 ± 11.3 (20–78) [*n* = 48]	48.3 ± 7.1 (35–57) [*n* = 10]	0.0055
SOC	52.9 ± 12.2 (30–77) [*n* = 47]	44.0 ± 6.1 (32–54) [*n* = 17]	
Baseline AHI^c^, /h—mean ± SD (range) [*n*]			
TH	37.8 ± 26.7 (5.3–110.0) [*n* = 48]	27.1 ± 17.4 (6.1–58.8) [*n* = 10]	0.0609
SOC	30.4 ± 15.9 (6.4–67.6) [*n* = 47]	23.5 ± 23.3 (6.9–99.8) [*n* = 17]	

^a^Based on maximum likelihood estimates in the multiple regression model

^b^Standard deviation

^c^Apnea-hypopnea index

### Program acceptance

The majority of TH patients (92.8 % [52/56]) stated that the new approach to monitoring met or exceeded their expectations compared with 94.5 % (52/55) in the SOC group (*p* = 0.82).

### Resource use

There was a significant reduction in the mean aggregate time required to coach a patient in the TH versus SOC group (Table 5). The number of follow-up clinic visits was similar in the two groups; only 17/58 patients (19 visits) in the TH group and 18/64 patients in the SOC group (20 visits) had a clinic visit. The mean total number of contacts was significantly lower in the TH group (2.2 ± 2.6/patient) compared with the SOC group (7.8 ± 4.1/patient; *p* < 0.0001), corresponding to a 59 % reduction in labor with the TH monitoring program. In CPAP-adherent patients, labor time was significantly lower in the TH versus SOC group (Table 5).

**Table 5:** Resource utilization based on patient contacts

	TH (*n* = 58)	SOC (*n* = 64)	*p* value
Total time utilized			
Contact attempt, minutes (4 min each)	116	920	
Follow-up contact, minutes (9 min each)	702	2214	
Clinic visit, minutes (30 min each)	570	600	
Total time for follow-up care, min	1388	3734	
Time for follow-up care per patient, min			
Mean±SD^a^ (range)	23.9 ± 26.3 (0–107)	58.3 ± 25.0 (18–133)	<0.0001
Median	18.0	52.5	
Time per patient—based on level of adherence, min			
Adherent patients, min	(*n* = 48)	(*n* = 47)	
Mean ± SD	20.7 ± 24.6	53.7 ± 20.8	<0.0001
Non-adherent patients, min	(*n* = 10)	(*n* = 17)	
Mean ± SD (*n*)	39.5 ± 29.6	71.2 ± 31.1	0.07

^a^Standard deviation

## Discussion

In this study, TH monitoring of CPAP adherence using the U-Sleep platform required 59 % less coaching labor than an SOC model, with equivalent adherence and efficacy. Adherence was achieved quickly, particularly in the TH group, nearly two thirds of whom had achieved Medicare-defined CPAP adherence within 30 days.

Previous studies examining the use of TH strategies for managing OSA patients have had varying results [13, 14, 17, 19, 21]. Few have explored resource utilization. The TELEPAP study was a randomized, controlled clinical study in 51 patients with newly diagnosed OSA [16]. The results did not show an advantage in adherence or efficacy for telemonitoring. With respect to labor requirements, it was assumed that standard clinical care was the lower cost option.

To our knowledge, there is only one other study documenting reduced labor requirements with TH [21]. A significant reduction in nursing time per patient in CPAP users managed using a wireless, remote monitoring system (ResTraxx Online System®; ResMed) compared with control was documented. DeMolles and colleagues used motivational interviewing techniques administered via an automated telephone-linked communication system to promote CPAP use [22]. CPAP use significantly increased in the telemedicine group, by 1 hour at 6 months and 2 hours at 12 months; labor costs were not assessed. In a 2-month pilot study in 45 newly diagnosed OSA patients, telemonitoring was as effective as usual care in achieving CPAP adherence and efficacy, but economic efficiency and labor requirements were not addressed [20]. Fox and colleagues randomized 75 patients in a 3-month study comparing SOC with a web-based telemonitoring application without automated messaging [23]. The telemedicine group had increased CPAP usage, and use of telemedicine was a significant predictor of CPAP adherence. However, on average, telemedicine patients required an extra hour of research coordinator time to achieve the improvement. The authors postulated that the additional labor cost associated with the TH approach might be worth the investment because of the improved adherence achieved.

In the current study, there was no significant difference in adherence between the TH and SOC groups. This may be because the overall CPAP adherence rate in the SOC group was at the high end of those reported in the literature. The study was designed to detect a 20 % difference in adherence between the SOC and TH groups, with an expected SOC group adherence rate of 70 %. To detect a significant difference between the two groups, adherence in the TH group would need to have been 90 %. Although the SOC techniques were clearly successful at obtaining adherence, they required significant time investment from HCPs, and this is expensive. We speculate that the decrease in resource use in the TH group can largely be attributed to a marked reduction in the requirement for HCPs to manually review adherence data, identify at-risk patients, and provide coaching. Instead, automated notifications were delivered to the patient and their care provider. The reduction in labor required in the TH group was especially evident in the savings associated with coaching patients who reached adherence within the first 30 days (Fig. 3). The TH program did not require any additional labor if the patient was responding well to CPAP therapy. In contrast, the SOC model required an intense labor investment, particularly during the first month.

The TH technology allowed personnel resources to be diverted to patients who were non-adherent with therapy or were having one of a defined set of clinical complications (e.g., increased AHI, high leak). The rule sets that trigger patient and/or provider notifications can be customized based on the specific requirements and scope of each healthcare provider and patient population. Patient satisfaction data indicated that the TH program met patient expectations just as effectively as the SOC program.

There are some limitations to this study. Firstly, the ability of the study to detect a significant difference in adherence and daily usage between the TH and SOC groups was reduced by the exclusion of 18 patients from the final analysis and the high adherence rate in the SOC group. Of the 18 patients with insufficient data, 12 were from the TH group and 6 were from the SOC group. Examination of the characteristics of these patients did not provide any explanation for the difference in dropout rates between the two groups, and there is no evidence that treatment allocation played any role. Secondly, the findings of this study may not be widely generalizable for the following reasons. Sleep Data’s SOC program utilizes frequent phone calls to follow and coach patients, which can be labor-intensive. Therefore, the coaching labor time saved with the U-Sleep program might be greater compared with less rigorous programs. In addition, the TH arm relied heavily on access to a phone network or the internet, which, although commonplace, is by no means universal. In particular, some of the most challenging patients are those in lower socioeconomic groups who do not have access to these communication tools. Thus, some patients may not have the ability to participate in a TH program. Thirdly, the time ascribed to each follow-up encounter was based on Sleep Data’s historical averages and not measured directly. It is possible that although the number of phone calls made to TH patients was markedly reduced, their duration might have been longer. Lastly, although there was close communication between Sleep Data and the prescribing physicians, CPAP therapy for all patients was primarily being managed by Sleep Data’s professionals; this study did not gather detailed information about interventions that might have been performed by the prescribing physician without Sleep Data’s knowledge.

This study also has a number of strengths. The protocol was designed to overcome some of the limitations of previous studies involving telemedicine and OSA. This study included more than one study center, enrolled a larger number of patients and utilized objective adherence data on which to base conclusions. In addition, the determination of resource utilization associated with TH versus SOC has not been widely analyzed previously. Finally, the TH system used, U-Sleep, is a HIPAA-compliant program that is currently available and provides a standardized model that can be applied in a variety of clinical environments to optimize treatment of OSA patients.

As healthcare payment structures in the USA continue to evolve, there is an increasing pressure on healthcare providers to focus on business efficiency, especially cost management. According to the Centers for Medicare and Medicaid Services, total US healthcare spending was $2.8 trillion in 2012, up 3.7 % versus 2011 [17]. Given such a heavy economic burden, more streamlined approaches to providing healthcare, such as TH, are necessary to maintain quality healthcare while reducing costs.

In conclusion, the U-Sleep TH intervention was associated with excellent adherence to CPAP therapy in newly diagnosed OSA patients. The U-Sleep program also significantly reduced the amount of HCP time that was needed to achieve adherence and efficacy comparable to the SOC model. Whether these benefits translate into better cost effectiveness remains to be determined.
